# Diet quality influences isotopic discrimination among amino acids in an aquatic vertebrate

**DOI:** 10.1002/ece3.1491

**Published:** 2015-04-25

**Authors:** Yoshito Chikaraishi, Shawn A Steffan, Yoshinori Takano, Naohiko Ohkouchi

**Affiliations:** 1 Japan Agency for Marine-Earth Science and Technology2-15 Natsushima-cho, Yokosuka, 237-0061, Japan; 2 USDA-ARS Vegetable Crops Research Unit, Department of Entomology, University of Wisconsin1630 Linden Dr., Madison, WI, USA

**Keywords:** Amino acids, diet quality, food web, nitrogen isotopic composition, trophic discrimination factor

## Abstract

Stable nitrogen isotopic composition of amino acids (*δ*^15^N_AA_) has recently been employed as a powerful tool in ecological food web studies, particularly for estimating the trophic position (TP) of animal species in food webs. However, the validity of these estimates depends on the consistency of the trophic discrimination factor (TDF; - Δ*δ*^15^N_AA_ at each shift of trophic level) among a suite of amino acids within the tissues of consumer species. In this study, we determined the TDF values of amino acids in tadpoles (the Japanese toad, *Bufo japonicus*) reared exclusively on one of three diets that differed in nutritional quality. The diets were commercial fish-food pellets (plant and animal biomass), bloodworms (animal biomass), and boiled white rice (plant carbohydrate), representing a balanced, protein-rich, and protein-poor diet, respectively. The TDF values of two “source amino acids” (Src-AAs), methionine and phenylalanine, were close to zero (0.3–0.5‰) among the three diets, typifying the values reported in the literature (∼0.5‰ and ∼0.4‰, respectively). However, TDF values of “trophic amino acids” (Tr-AAs) including alanine, valine, leucine, isoleucine, and glutamic acid varied by diet: for example, the glutamic acid TDF was similar to the standard value (∼8.0‰) when tadpoles were fed either the commercial pellets (8.0‰) or bloodworms (7.9‰), but when they were fed boiled rice, the TDF was significantly reduced (0.6‰). These results suggest that a profound lack of dietary protein may alter the TDF values of glutamic acid (and other Tr-AAs and glycine) within consumer species, but not the two Src-AAs (i.e., methionine and phenylalanine). Knowledge of how a nutritionally poor diet can influence the TDF of Tr- and Src-AAs will allow amino acid isotopic analyses to better estimate TP among free-roaming animals.

## Introduction

**Figure 1:**
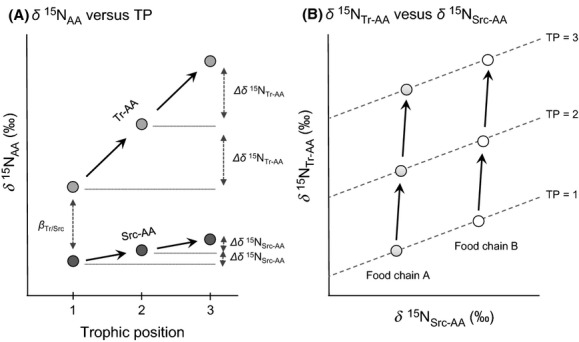
Schematic illustration of (A) the relationship between the *δ*^15^N values of trophic and source amino acids (Tr-AA and Scr-AA, respectively) and the trophic position of organisms in food webs (TDF_T__r-__AA_ - Δ*δ*^15^N_TR__-__AA_, TDF_S__rc-__AA_ - Δ*δ*^15^N_src-__AA_, and TDF_T__r/Scr_ - TDF_T__r-__AA_ − TDF_S__rc-__AA_, after Chikaraishi et al. 2007, 2009) and (B) the cross-plot for the *δ*^15^N values of Tr- and Scr-AAs for consumer and resource species in two food chains with different background of the *δ*^15^N values (dashed lines indicate the trophic isocline for integer-based number TPs, 1.0, 2.0, and 3.0 based on the eq. 1, after Chikaraishi et al. 2014).

Stable nitrogen isotopic composition of amino acids (*δ*^15^N_AA_) has recently been employed as a new method in ecological food web studies, particularly for understanding trophic linkage and energy flow among animal species in complex ecosystems, as well as estimation of background isotopic signals within and among habitats (e.g., McCarthy et al. 2007; Popp et al. 2007; Chikaraishi et al. 2009, 2014). This method has been constructed based on the contrasting isotopic discrimination (Δ*δ*^15^N) associated with “source amino acids” (Src-AAs; e.g., methionine and phenylalanine) and “trophic amino acids” (Tr-AAs; e.g., alanine, valine, leucine, isoleucine, proline, and glutamic acid) (Fig.1). Tr-AAs tend to show significant ^15^N-enrichment by ∼3–8‰ during the transfer of amino acids from one trophic level to another (Δ*δ*^15^N_Tr-AA_ in Fig.1A). Because the metabolism of amino acids begins with transamination or deamination, which always cleaves carbon–nitrogen bonds, there is the potential for discrimination between heavy (^15^N) and light (^14^N) isotopes (Chikaraishi et al. 2007; Ohkouchi et al. 2015). Conversely, Src-AAs show little ^15^N-enrichment by ∼0–1‰ (Δ*δ*^15^N_Src-AA_ in Fig.1A) because their initial metabolic steps are generally dominated by reactions that neither form nor cleave carbon–nitrogen bonds (Chikaraishi et al. 2007). Thus, the isotopic composition of Src-AAs in consumers principally reflects an integrated value for the isotopic composition of diets. The difference between Tr- and Src-AAs within a single organism corresponds to the TP of the organism (e.g., McCarthy et al. 2007; Popp et al. 2007; Chikaraishi et al. 2009, 2014). This phenomenon permits the empirical assembly of trophic hierarchies within food webs, independent of background variation in *δ*^15^N values (Steffan et al. 2013; Chikaraishi et al. 2014). On the cross-plot for the *δ*^15^N_Tr-AA_ and *δ*^15^N_Src-AA_ values (Fig.1B), resource and consumer species in a single food chain tend to be arrayed in an approximately vertical column, aligned with the integrated *δ*^15^N_Src-AA_ value of the basal resource of the food chain. For each integer-based trophic level, pairings of *δ*^15^N_Tr-AA_ and *δ*^15^N_Src-AA_ values create a trophic isocline (also referred to as a *trophocline*, Chikaraishi et al. 2014). The trophocline of any given trophic position (TP_*x*/*y*_) is defined by the following equation, derived originally from Chikaraishi et al. (2009):

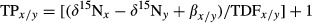
1where *x* denotes “trophic” amino acids; *y* denotes “source” amino acids; *β*_*x*/*y*_ represents the isotopic difference between trophic and source amino acids in primary producers (e.g., phytoplankton or higher plants) at the base of a food web; and TDF_*x*/*y*_ represents the net intertrophic ^15^N-enrichment between a trophic and source amino acid (TDF_*x*/*y*_ - TDF_*x*_ − TDF_*y*_). The TDF_*x*_ (or TDF_*y*_) value represents the change in ^15^N for a single amino acid at each trophic transfer (i.e., TDF_Tr-AA_ - Δ*δ*^15^N_Tr-AA_ and TDF_Scr-AA_ - Δ*δ*^15^N_Scr-AA_ in Fig.1A). Chikaraishi et al. (2009, 2010a) established the importance of glutamic acid and phenylalanine to serve, respectively, as amino acids *x* (- glutamic acid, the most predictable Tr-AA) and *y* (- phenylalanine, the most predictable Src-AA), for accurate and precise estimates of animal trophic position (Chikaraishi et al. 2007, 2009, 2011; Steffan et al. 2013). Several studies have used weighted mean isotopic composition of Tr- and Src-AAs instead of *δ*^15^N_Glu_ and *δ*^15^N_Phe_ values (Sherwood et al. 2011; Vander Zanden et al. 2013). Such groupings have been proposed as a means of providing greater statistical power than focusing on glutamic acid and phenylalanine alone (Décima et al. 2013), but evidence of increased accuracy or precision in TP assignment deriving from grouping Tr- and Src-AAs has yet to be demonstrated.

The validity of the TP estimate is principally dependent on the consistency of both *β* and TDF values, which have been evaluated based on relatively small number of cultured (i.e., controlled feeding experiments) and well-characterized wild species (e.g., caterpillars on leaves, predaceous insects) so far (Chikaraishi et al. 2009, 2011; Steffan et al. 2013). For example, McClelland and Montoya (2002) first reported 6.7 ± 0.6‰ and 0.3 ± 1.1‰ for the TDF values of glutamic acid (TDF_Glu_) and phenylalanine (TDF_Phe_), respectively, in cultured zooplankton (*N* - 2); Chikaraishi et al. (2009) measured the respective TDF_Glu_ and TDF_Phe_ values as 8.0 ± 1.2‰ and 0.4 ± 0.5‰ using 10 independent samples of cultured zooplankton, fish, and gastropods. Thus, altogether, 12 samples were used to provide the initial estimates of *β* and TDF (Chikaraishi et al. 2009). More recently, Steffan et al. (2013) confirmed that the TDF values were not only consistent with previously reported value (i.e., 8.0 ± 0.4‰ for glutamic acid and 0.4 ± 0.3‰ for phenylalanine), but were also constant at trophic levels 3 and 4 (higher-order carnivores) in controlled feeding experiments involving two different insect communities (N - 4 for each trophic level). Bradley et al. (2014) calculated 7.8 ± 0.2‰ and 1.5 ± 0.3‰ for the respective TDF_Glu_ and TDF_Phe_ values in long-term controlled feeding experiments involving the Bluefin tuna *Thunnus orientalis*, a top carnivore. Thus, the TDF value obtained from cultured and diet-known species seems to be relatively consistent, particularly for glutamic acid and phenylalanine among a wide range of taxa and trophic levels, including zooplankton, gastropods, fish, and insects. In fact, the TDF values reported in Chikaraishi et al. (2009) have been used to provide highly resolved images of complex food webs, representing a broad range of trophic positions (i.e., 1–5) and a total of 89 species from coastal marine and terrestrial ecosystems (Steffan et al. 2013; Chikaraishi et al. 2014).

However, the universality of the TDF values has been questioned for several wild animals including penguins (Lorrain et al. 2009), large carnivorous fish (Dale et al. 2011; Hoen et al. 2014), sea turtles (Seminoff et al. 2012), jumbo squids (Ruiz-Cooley et al. 2013), and harbor seals (Germain et al. 2013). For example, Dale et al. (2011) reported that the TP_Glu/Phe_ values of wild brown stingrays were underestimated by ∼1 unit (9 of 11 of stingrays had TP_Glu/Phe_ < 3.0) compared with the trophic position independently estimated by stomach content analysis. Also, Germain et al. (2013) reported a significant compression to 2.7 ± 1.9‰ for the TDF_Glu_ value (but no compression for the TDF_Phe_ value, −0.8 ± 0.3‰) in harbor seals relative to a potential food source (i.e., herring). If the TDF_Glu/Phe_ value (- TDF_Glu_ − TDF_Phe_) is reduced from 7.6‰ to 3.5‰ in wild animals, the TP_Glu/Phe_ value calculated from the eq. 1 is increased by approximately twice for studied consumers. These results may suggest that the TDF values of amino acids in wild, free-roaming animals are somehow different from those found in cultured and/or well-characterized species. This is unlikely the case for the zooplankton or fish cultured by Chikaraishi et al. (2009), or the insect communities cultured by Steffan et al. (2013), given that these consumers were provided ecologically realistic diets. Nevertheless the question deserves further investigation, particularly involving greater breadth of vertebrate and microbial species. Several previous studies (e.g., Hobson et al. 1993; Robbins et al. 2005; Tsahar et al. 2008) have reported the effect of the dietary protein quality on the TDF values of bulk tissue of consumer species. Dietary protein quality, content, trophic position, and/or the form of nitrogen excretion have been speculated to be determinants of amino acid TDFs (Germain et al. 2013; Hoen et al. 2014), but again, evidence is lacking. Thus, the factors affecting the TDF of amino acids in consumer species should be further examined using homogeneous, well-characterized diets, to better understand the trophic ecology of species in natural food webs.

**Figure 2:**
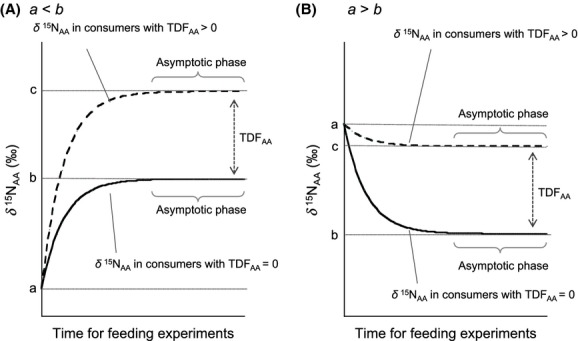
Mass balance models for the change in the *δ*^15^N values of amino acid during controlled feeding experiments, in the case of (A) a < b, and (B) a > b: a - *δ*^15^N _AA_ in consumers before feeding, b  -  *δ*^15^N _AA_ in diets used in the feeding experiments, and c - b + TDF_AA_. Solid and dashed lines indicate the *δ*^15^N _AA_ value in consumers with the TDF_AA_ - 0 and >0, respectively.

In this study, we determined the effect of diet quality on the TDF value of amino acids, based on controlled feeding experiments involving larvae (tadpoles) of the Japanese toad, *Bufo japonicus*, reared on three distinct diets: commercial pellets, bloodworms, and boiled rice, all of which represented a range of protein and carbohydrate concentrations. The commercial pellets provided a balanced mixture of protein (>30%), carbohydrate (<5%), lipids (<4%), lime (<12%), and other nutrients, while the bloodworms were largely a protein- and lipid-rich diet. The boiled rice was protein-depleted, but carbohydrate-rich. We used tadpoles as a model organism in the controlled feeding trials because they can develop on a variety of diets (from boiled rice to bloodworms). As the tadpoles grow under these diet regimens, it is expected that their biomass nitrogen will gradually be replaced by diet-derived proteins, accompanied with exponential to asymptotic changes in the *δ*^15^N value of amino acids during feeding experiments (Fig.2). Thus, the TDF values can be obtained by the difference in the *δ*^15^N value of the amino acids between tadpoles and their diets in the asymptotic phase of the feeding experiments.

## Materials and Methods

### Animal rearing

**Figure 3:**
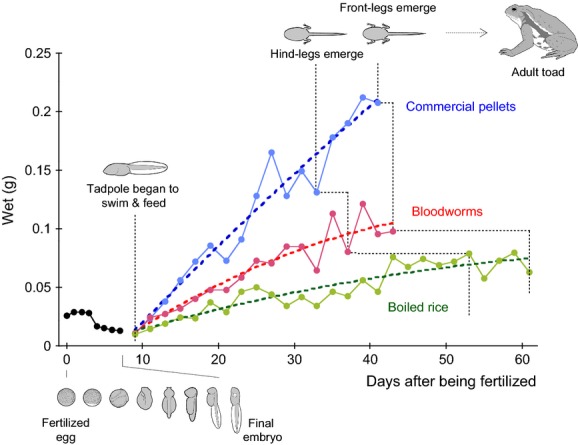
Growth carves of tadpoles feeding on (blue) artificial pellets, (red) bloodworms, or (green) boiled rice. Filled circle indicates measured weight (g) of wet biomass. Nine days after being fertilized, the embryo matured into a young tadpole, and began to feed on one of three diets: commercial fish-food pellets, bloodworms, or boiled rice.

Newly fertilized eggs of the Japanese toad, *Bufo japonica*, were collected from a garden pond in Yugawara, Japan (35°08′N, 139°07′E), and reared in de-chlorinated water at room temperature (22–25°C). As shown in Fig.3 9 days after being fertilized, the embryo matured into a young tadpole, and began to swim and feed on one of three diets: commercial fish-food pellets, bloodworms, or boiled rice. These diets were stored at −20°C before feeding, and excess amounts of the fresh diets were provided to the tadpoles every morning and evening after changing the reared water to remove uneaten food. All of tadpoles swam around actively, and no tadpole died during the feeding experiments. Hind-leg development in the tadpoles occurred at 33, 37, and 53 days, and front legs emerged at 41, 43, and 61 days after being fertilized, when the tadpoles fed on commercial pellets, bloodworms, and boiled rice, respectively. Thus, all tadpoles grew and developed during the experiments (i.e., there was no starvation), and these growth rates were faster than those of wild tadpoles, which generally requires 80–90 days for similar levels of development, but this may be attributable to the lack of wind, rain, temperature fluctuations, or predation threats, all of which represent stressors for tadpoles. Two individuals were sampled every day during 0–7 days and every two days during 7–61 days from each diet treatment. The sampled tadpoles were cleaned with tap water and stored at −20°C immediately.

### Amino acid analysis

For the fertilized egg (0 day) and the late stage embryo (7 days), whole samples were analyzed for their amino acid isotopic values. For hatched tadpoles (9–61 days), only the tails of tadpoles were used (to minimize possible contamination from the diet or excrement). For this analysis, we prepared single individuals from each sample for molecular isotope analysis of amino acids, to evaluate the replacement of biomass proteins, during the period of time characterized by both exponential and asymptotic changes in *δ*^15^N values (Fig.2). Then, after isolating the period of time characterized by minimal (- asymptotic) changes in *δ*^15^N values, we prepared samples only from this period. This period represented the last 20 days before the front legs of the tadpoles had emerged. We calculated the TDF values for each diet based on 10 data points during this asymptotic phase of the feeding experiments.

The samples were prepared for the molecular isotope analysis after HCl hydrolysis and *N*-pivaloyl/isopropyl (Pv/iPr) derivatization, according to the procedure in Chikaraishi et al. (2009, 2010b). In brief, the samples were hydrolyzed using 12 mol/L HCl at 110°C. The hydrolysate was washed with *n*-hexane/dichloromethane (3/2, v/v) to remove hydrophobic constituents. Then, derivatizations were performed sequentially with thionyl chloride/2-propanol (1/4) and pivaloyl chloride/dichloromethane (1/4). The abundance of amino acids was determined by gas chromatography (GC) using a 6890N GC instrument connected to the flame ionization detector (FID) and nitrogen–phosphorus detector (NPD) (Agilent Technologies, Palo Alto, CA). The Pv/iPr derivatives of amino acids were injected using a programmable temperature vaporizing (PTV) injector (Gerstel, Mülheim, Germany) into a VF-35 ms capillary column (30 m; i.d. 0.52 mm; film thickness 0.50 *μ*m; Agilent Technologies). The carrier gas (He) flow rate was controlled using a constant flow mode at 6.0 mL min^−1^. The abundance of 11 amino acids (alanine, glycine, valine, leucine, isoleucine, threonine, aspartic acid, serine, methionine, glutamic acid, and phenylalanine) was quantified by comparison with the peak area of NPD chromatograms with external amino acid references. These amino acids were chosen because they have relatively constant recovery (<±10%) among replicate analyses in this method. Stable nitrogen isotopic composition of amino acids was determined by gas chromatography/isotope ratio mass spectrometry (GC/IRMS) using a 6890N GC (Agilent Technologies) instrument coupled to a Delta^plus^XP IRMS instrument through combustion (950°C) and reduction (550°C) furnaces, countercurrent dryer (Permeable membrane, Nafion™), and liquid nitrogen CO_2_ trap via a GC-C/TC III interface (Thermo Fisher Scientific, Bremen, Germany). The Pv/iPr derivatives of amino acids were injected using a PTV injector (Gerstel) into an HP Ultra-2 capillary column (50 m; i.d. 0.32 mm; film thickness 0.52 *μ*m; Agilent Technologies). The carrier gas (He) flow rate was controlled using a constant flow mode at 1.4 mL min^−1^. To assess the reproducibility of the isotope measurement and to obtain the isotopic composition, reference mixtures of 9 amino acids (alanine, glycine, leucine, norleucine, aspartic acid, methionine, glutamic acid, phenylalanine, and hydroxyproline) with known *δ*^15^N values (ranging from −26.4‰ to +45.6‰, Indiana University, Bloomington, IN, SI Science Co., Sugito-machi, Japan; Sato et al. 2014) were analyzed after every five to eight samples runs, and three pulses of reference N_2_ gas were discharged at the beginning and end of each chromatography run for both reference mixtures and samples. The isotopic composition of amino acids in samples was expressed relative to atmospheric nitrogen (air) on scales normalized to known *δ*^15^N values of the reference amino acids. The accuracy and precision for the reference mixtures were always 0.0‰ (mean of Δ) and 0.4–0.7‰ (mean of 1*σ*) for sample sizes of ≥1.0 nmol N, respectively. The *δ*^15^N values of the following 8 amino acids: alanine, glycine, valine, leucine, isoleucine, glutamic acid, and phenylalanine were determined for all samples, but methionine was determined for approximately half of the samples due to its small amount in the samples (Table1), based on the S/N ratio of ≥20 with baseline separation on the chromatogram.

**Table 1:** Stable nitrogen isotopic composition (δ^15^N value) of amino acids in the three diets and in tadpoles before and after feedings

Days after being fertilized	δ^15^N (‰, relative to Air)							
	Alanine	Glycine	Valine	Leucine	Isoleucine	Methionine	Glutamic acid	Phenylalanine
Diets								
Commercial pellets	15.9	4.4	15.3	13.1	11.7	3.0	13.4	7.3
Bloodworms	15.9	7.1	17.3	7.2	7.4	2.7	19.1	6.8
Boiled rice	4.7	−0.1	3.8	2.4	0.7	−3.7	2.8	10.1
Tadpole before feeding								
0	14.9	7.5	15.1	10.9	10.9	0.2	14.6	4.6
7	16.3	2.7	16.4	10.4	17.3	0.4	16.4	4.3
9	16.3	3.9	18.2	11.9	19.3	0.3	17.2	4.7
Tadpole feeding on commercial pellets								
11	21.5	8.7	18.9	13.6	19.5	n.d.	20.2	7.0
15	22.6	12.5	19.8	14.4	19.0	3.1	21.2	7.1
19	23.0	9.5	20.5	14.4	19.3	n.d.	22.2	7.8
23 #1	23.2	9.7	21.4	14.1	19.6	3.5	21.3	7.4
23 #2	23.3	8.4	21.9	15.7	18.9	2.3	22.1	7.3
27 #1	20.0	8.6	20.3	16.0	18.9	3.5	20.7	7.0
27 #2	24.0	11.9	21.9	14.7	18.1	n.d.	20.8	7.9
31 #1	23.9	12.5	21.1	15.1	19.3	n.d.	21.7	7.0
31 #2	23.8	10.6	22.6	15.8	20.1	4.1	21.1	7.9
35 #1	24.3	10.5	22.7	15.1	19.1	3.1	21.2	7.5
35 #2	23.6	9.7	22.7	15.0	19.5	n.d.	21.4	7.4
39 #1	23.7	11.4	23.4	14.8	19.5	n.d.	22.1	7.6
39 #2	23.4	9.8	22.5	14.7	19.5	n.d.	21.8	7.9
Tadpole feeding on bloodworms								
11	17.6	6.1	21.2	13.2	16.5	n.d.	25.6	6.8
15	18.6	8.4	22.2	13.9	15.9	n.d.	26.6	6.9
19	19.6	12.2	21.4	13.5	14.5	n.d.	26.7	7.0
23 #1	21.0	10.1	21.6	13.1	15.4	3.8	27.2	6.9
23 #2	21.3	9.7	21.9	13.4	14.6	3.8	26.9	7.4
27 #1	20.1	8.3	22.2	12.7	14.9	n.d.	26.5	7.1
27 #2	20.9	9.7	21.4	12.8	15.1	n.d.	27.4	6.7
31 #1	20.9	10.2	21.2	13.3	14.8	2.7	26.8	7.1
31 #2	20.2	11.4	22.2	13.1	14.5	n.d.	26.9	7.0
35 #1	21.2	9.1	21.9	12.3	13.5	2.4	27.2	7.2
35 #2	20.8	8.5	22.1	13.3	14.6	n.d.	26.8	7.3
39 #1	21.3	12.6	22.5	12.6	13.7	n.d.	26.9	7.3
39 #2	20.5	8.4	21.9	13.7	14.6	3.1	26.9	7.3
Tadpole feeding on boiled rice								
11	11.8	1.6	12.8	7.4	13.8	−2.4	12.7	7.2
15	10.4	3.5	11.0	6.0	10.2	n.d.	9.5	9.0
19	9.7	1.6	9.7	5.6	8.7	n.d.	8.4	9.8
23	9.2	1.4	9.4	6.2	7.1	n.d.	5.3	9.5
27	9.6	0.3	9.4	5.0	6.3	−3.3	4.0	10.6
31	8.8	−0.2	8.3	4.5	6.4	−3.9	4.8	9.9
35	9.0	−0.2	7.0	5.6	5.1	n.d.	4.7	10.3
39	9.7	2.0	7.0	4.8	5.3	n.d.	4.1	10.8
43 #1	8.6	0.2	7.7	4.6	4.5	−3.2	4.1	10.7
43 #2	8.5	−1.4	7.3	4.4	5.5	−3.7	3.5	10.7
47 #1	8.3	−0.3	7.9	4.5	5.1	n.d.	4.1	10.5
47 #2	9.1	−0.4	7.9	4.2	4.8	−3.5	4.4	10.4
51 #1	8.6	−1.0	7.1	4.9	4.6	−3.4	3.5	11.0
51 #2	7.9	−0.7	7.8	4.4	4.4	−3.3	3.1	10.7
55 #1	7.5	0.2	7.1	4.7	4.2	−3.2	3.7	10.9
55 #2	7.9	−0.7	7.3	4.4	4.7	−3.4	2.9	10.6
59 #1	7.9	0.7	7.3	3.9	4.6	n.d.	3.6	10.5
59 #2	8.1	−0.5	7.1	4.0	4.9	−3.0	3.2	10.7

For each feeding experiment, the TDF values of the 8 amino acids were calculated based on the measurements during the asymptotic phase (i.e., the last 20 days before the front legs had emerged) of the *δ*^15^N dynamic during feeding experiments. For all diets, the asymptotic period began by day 23. For the commercial pellet and bloodworm diets, there were five separate tadpole collections (on days 23, 27, 31, 35 and 39) spanning day 23 to day 39 (- emergence of front legs). With the boiled rice diet, the period of time required to reach the front-leg emergence stage was much longer (day 59). The 5 separate tadpole collections, therefore, had to be made on days 43, 47, 51, 55 and 59. Mean *δ*^15^N value and 1*σ* for each amino acid in each diet regimen are reported.

### Statistical analysis

The effect of tadpole diet (commercial fish pellets, bloodworms, and boiled rice) on the TDF value for each amino acid was assessed using one-way analysis of variance (SigmaPlot 12.3 Systat Software Inc, San Jose, CA). One-sample *t*-tests were used test whether the TDF values of Src-AAs (phenylalanine, methionine, and glycine) diverged significantly from zero.

## Results and Discussion

### Amino acid composition

**Figure 4:**
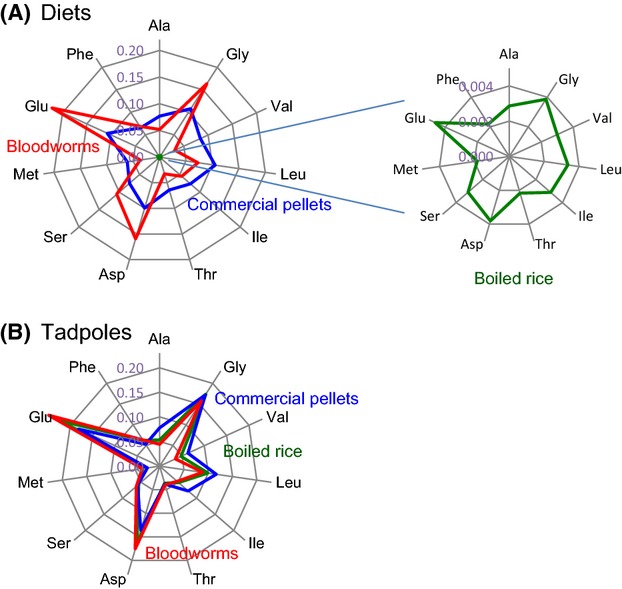
Molar ratios of 11 amino acids for (A) three diets (relative to sum of bloodworm - 1) and for (B) tadpoles on average the last 20 days before their front legs had emerged (relative to sum - 1).

The abundance and molecular profile of amino acids were significantly different among the three diets (Fig.4). The commercial pellets are characterized by relatively homogeneous composition of the 11 amino acids (∼48 ± 14 mg g^−1^ for each). The bloodworms are characterized by heterogeneous amino acid composition as a large amount of glutamic acid, glycine, and aspartic acid (153, 58, and 100 mg g^−1^, respectively) but a small amount of valine, threonine, alanine, and methionine (17, 18, 22, and 25 mg g^−1^, respectively). The boiled rice is composed mainly of starch and therefore contains a quite small amount of amino acids (relatively homogenous ∼1.8 mg g^−1^ for each of 11 amino acids). The detected 11 amino acids account for 53, 58, and 2 wt. % of the commercial pellets, bloodworms, and boiled rice, respectively. On the other hand, the relative abundances of the amino acids were quite similar among tadpoles even though they fed on the different diets: proportionally large amounts of glutamic acid, glycine, and aspartic acid, but small amounts of valine, threonine, alanine, and methionine (Fig.4).

### Change in δ^15^N value during 0–9 days (from egg to tadpole before feeding)

The ^15^N-enrichment of tadpole biomass occurred prior to the start of the feeding trial (Table1). From 0 day (egg stage) to 9 day (young larva, immediately before feeding began), there was a relatively large degree of ^15^N-enrichment (+8.4‰) in isoleucine and relatively small ^15^N-enrichment in alanine (+1.4‰), valine (+3.1‰), leucine (+0.9‰), and glutamic acid (+2.7‰) (Table1). In contrast, ^15^N-depletion (by −3.6‰) occurs in glycine, and little change in *δ*^15^N value is found in methionine (by +0.2‰) and phenylalanine (by ±0.0‰). Except for isoleucine and glycine, the high ^15^N-enrichment of Tr-AAs and low enrichment in Src-AAs from egg to tadpole (before feeding) mirrors the trends in the trophic isotopic discrimination in typical grazing food webs (e.g., McCarthy et al. 2007; Popp et al. 2007; Chikaraishi et al. 2009), implying that trophic isotopic discrimination occurs from yolk to embryo during embryonic processes in animals. This is consistent with the decrease in the body mass of tadpoles from 0 to 9 days of the experiments (Fig.3). The relatively small degrees of enrichment, particularly for alanine, leucine, and glutamic acid, are probably explained by the fact that these samples are an admixture of the ^15^N-enriched amino acids in the embryo and remaining yolk-derived (i.e., nonenriched, original) amino acids. However, it has not been reported previously that there is a large ^15^N-enrichment in isoleucine and ^15^N-depletion in glycine.

### Change in δ^15^N value of Src-AAs during feeding

**Figure 5:**
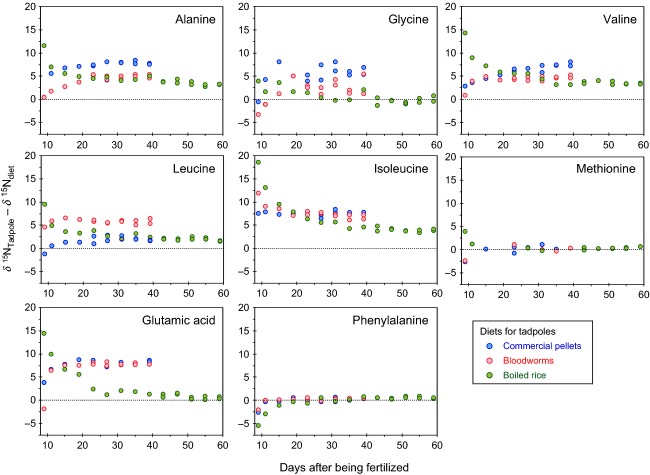
Difference in the *δ*^15^N value between tadpoles and diets: (blue) commercial pellets, (red) bloodworms, and (green) boiled rice, with respect to the days after being fertilized.

Across the three controlled feeding experiments, the *δ*^15^N values of two representative Src-AAs, methionine and phenylalanine, gradually converged on the *δ*^15^N values within the diets (Fig.5), plateauing at what appeared to be an asymptotic level. For methionine, mean (± 1*σ*) TDF values of tadpoles feeding on pellets, bloodworms, and rice were 0.3 ± 0.7‰, 0.5 ± 0.6‰, and 0.3 ± 0.2‰, respectively. Among the three diets, the TDF_Met_ values were not significantly different from one another (*F*_2,14_ - 0.174, *P *-* *0.842), nor was there significant evidence that any of the TDF_Met_ values diverged from zero (pellets: *t*_4_ - 1.10, *P *-* *0.332; bloodworms: *t*_4_ - 1.71, *P *-* *0.082; rice: *t*_4_ - 2.71, *P *-* *0.054). The Src-AA, phenylalanine, produced TDF_Phe_ values of 0.4 ± 0.3‰, 0.4 ± 0.3‰, and 0.5 ± 0.1‰ for tadpoles feeding on pellets, bloodworms, and rice diets, respectively. Across the three diet types, the TDF_Phe_ values were not significantly different from one another (*F*_2,14_ - 0.505, *P *-* *0.616). The TDF_Phe_ values were all near zero, and while the pellet diet was not different from zero (pellets: *t*_4_ - 2.61, *P *-* *0.060), the TDF_Phe_ values of the other two diets were bloodworms: *t*_4_ - 2.95, *P *-* *0.042 and rice: *t*_4_ - 8.99, *P *<* *0.001. For both methionine and phenylalanine, therefore, the impact of diet on the TDF was insignificant. Additionally, their respective TDFs reflect a relatively small degree of ^15^N-enrichment, which is consistent with previously reported TDF values for Src-AAs (∼0.5–0.6‰ and ∼0.4–1.5‰ for methionine and phenylalanine, respectively; Table2). Altogether, these results suggest that for this amphibian species, diet quality has a trivial impact on the TDF values of the two Src-AAs (methionine and phenylalanine).

**Table 2:** The trophic discrimination factor (TDF) observed in the present study and literatures

	The present study											
	Commercial pellets		Bloodworms		Boiled rice		Chikaraishi et al. (2009)		Steffan et al. (2013)		Bradley et al. (2014)	
	TDF	1*σ*	TDF	1*σ*	TDF	1*σ*	TDF	1*σ*	TDF	1*σ*	TDF	1*σ*
Alanine	7.4	1.2	5.0	0.4	3.5	0.5	6.1	2.1	6.0	0.8	6.8	0.9
Glycine	5.7	1.3	2.4	1.2	−0.6	0.4	3.7	3.9	3.9	4.6	3.4	0.2
Valine	6.8	0.9	4.6	0.4	3.6	0.3	5.0	1.7	5.9	0.6	2.3	0.3
Leucine	2.1	0.6	5.8	0.4	2.0	0.3	4.8	2.0	3.8	0.4	7.1	0.7
Isoleucine	7.6	0.5	7.2	0.6	4.0	0.4	4.8	1.7	4.1	0.8	n.d.	n.d.
Methionine	0.3	0.7	0.5	0.6	0.3	0.2	0.5	0.6	0.6	0.3	n.d.	n.d.
Glutamic acid	8.0	0.5	7.9	0.3	0.6	0.6	8.0	1.2	8.0	0.3	7.8	0.2
Phenylalanine	0.4	0.3	0.4	0.3	0.5	0.1	0.4	0.5	0.4	0.2	1.5	0.3

The *δ*^15^N values of glycine, however, varied among the three controlled feeding experiments, as well as within a single feeding experiment (Fig.5). The magnitude of the enrichment (TDF_Gly_) differed significantly between diets (*F*_2,14_ - 44.23, *P *<* *0.001), registering TDF_Gly_ values of 5.7 ± 1.2, 2.4 ± 1.2‰, and −0.6 ± 0.3, when the tadpole fed on commercial pellets, bloodworms, and boiled rice, respectively (Table2). Each of the diets produced TDF_Gly_ values that were significantly different from zero (pellets: *t*_4_ - 9.74, *P *<* *0.001; bloodworms: *t*_4_ - 4.38, *P *-* *0.012; rice: *t*_4_ - −3.75, *P - *0.020), demonstrating that the three diets produced very different degrees of ^15^N enrichment in the glycine of the tadpoles, and the enrichment was always nonzero. In previous studies, glycine has frequently been classified among the Src-AAs (e.g., Popp et al. 2007; Sherwood et al. 2011; Vander Zanden, 2013), because the TDF_Gly_ value was often close to zero in zooplankton (0.7 ± 0.6‰; McClelland and Montota, 2002; Chikaraishi et al. 2009). However, the substantial, positive TDF_Gly_ values in our current study involving an amphibian, as well as past studies involving fish (Chikaraishi et al. 2009) and insects (Chikaraishi et al. 2011, 2014; Steffan et al. 2013), suggest that glycine may not actually be a Src-AA (Table2).

### Change in δ^15^N value of Tr-AAs from tadpole during feeding

The *δ*^15^N value of the five Tr-AAs (alanine, valine, leucine, isoleucine, and glutamic acid) in tadpoles changed over time (Fig.5), becoming asymptotic over the last 20 days of the feeding experiments and converging near the dietary value. The TDF_Glu_ value of glutamic acid, typically the most sensitive Tr-AA (Chikaraishi et al. 2009), was strongly affected by the diet type eaten by tadpoles. While the TDF_Glu_ values were very similar between the commercial pellets (8.0 ± 0.5) and the bloodworms (7.9 ± 0.3‰) (*t *-* *0.36, *P *-* *0.723), these were both profoundly different from the TDF_Glu_ of boiled rice (*F*_2,14_ - 409.16, *P *<* *0.001), which was 0.6 ± 0.6‰. The TDF_Glu_ values of pellets and bloodworms are consistent with the currently available TDF_Gly_ value (∼7–9‰) reported previously (Table2). However, when fed the rice diet, the *δ*^15^N values of glutamic acid in the tadpoles gradually converged on the *δ*^15^N values in the rice. The TDF_Glu_ value is thus surprisingly small (0.6 ± 0.6‰). Such a small TDF_Glu_ has never been reported previously in controlled feeding experiments for zooplankton, fish, gastropods, or insects (Table2). The effect of diet type on TDF was also highly significant for the other Tr-AAs, such as alanine (*F*_2,14_ - 142.97, *P *<* *0.001), valine (*F*_2,14_ - 118.70, *P *<* *0.001), leucine (*F*_2,14_ - 189.65, *P *<* *0.001), and isoleucine (*F*_2,14_ - 68.67, *P *<* *0.001). The magnitude of these effects varied among amino acids, but there was a distinct trend (TDF_Commercial pellets_ > TDF_Bloodworms_ > TDF_Boiled rice_) across most of the Tr-AAs. These data suggest that a diet profoundly lacking in protein, such as boiled rice, can influence the TDF values of Tr-AAs in consumer species.

### Effect of diet quality on the TDF value

The magnitude of intertrophic isotopic discrimination of amino acids in consumer species is likely mediated by the following: (1) whether amino acid metabolism begins with the cleaving/formation of nitrogen bonds, or (2) isotope effect and flux of the initial step (Chikaraishi et al. 2007; Ohkouchi et al. 2015). The initial steps of metabolism for methionine and phenylalanine are generally dominated by the formation of S-adenosylmethionine and tyrosine, respectively, which neither form nor cleave the carbon–nitrogen bond (Bender 2002). Thus, there is no opportunity for discrimination among the nitrogen isotopes in methionine and phenylalanine, thereby preempting ^15^N-enrichment, and suppressing TDF value (∼0‰) for these amino acids, even if the consumer species has fed on diets differing in quality (Table2).

**Figure 6:**
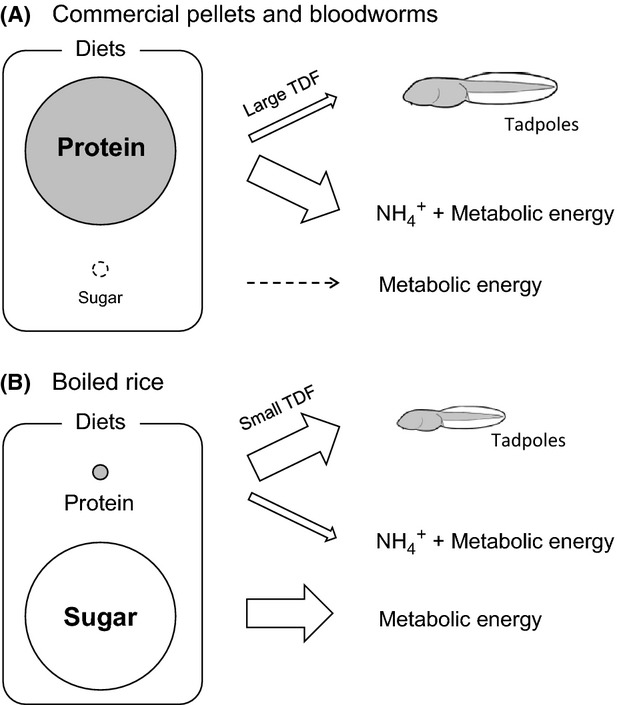
Possible metabolic flux of amino acids and sugar between tadpoles and diets, with respect to the metabolic energy production and the TDF value of TrAAs (and also glycine in the present study): (A) commercial pellets and bloodworms are mainly composed of proteins, and a large portion of the proteins is metabolized as major resources of metabolic energy while a remaining small portion is used for biomass construction if the tadpoles feed on either commercial pellets or bloodworms; and (B) boiled rice is mainly composed of sugars, and the sugars are metabolized as major resources of metabolic energy, while rice proteins are used mainly for biomass construction if the tadpoles feed on boiled rice. Size of circles indicates relative mass of proteins and sugars in the diets. Arrow size indicates the relative metabolic flux to energy and protein production.

In contrast, the other six amino acids investigated (i.e., five TrAAs: alanine, valine, leucine, isoleucine, and glutamic acid as well as one Src-AA: glycine) have transamination or deamination as a dominant or subdominant initial step of the amino acid metabolisms (Bender 2002). During these processes, a large portion of the amino acid “population” is deaminated to form ammonia (and keto acids), after consumers assimilate the amino acids from dietary protein (Fig.6). This would be quite important as a major process not only for the production of energy in consumer species but also for the discrimination of the nitrogen isotopes, where the preferential ^15^N-depletion on ammonia and ^15^N-enrichment on the remained amino acids occur theoretically (Chikaraishi et al. 2007). In fact, Miura and Goto (2012) estimated the isotopic discrimination factor (*α* - 0.9958) for an enzymatic transamination of glutamic acid to form aspartic acid in vitro and calculated that the TDF_Glu_ value of 8.0‰ corresponds to 86% of glutamic acid being deaminated and the remaining 14% of glutamic acid being used for biomass creation. The magnitude of ^15^N-enrichment on the remaining amino acids thus depends on the metabolic flux of deamination, maximizing the probability that most amino acids are deaminated. However, it is also hypothesized that minimal ^15^N-enrichment of the remaining amino acids in biomass is possible when the consumer species can derive metabolic energy from other resources, such as sugar and lipids but not amino acids (- amino acids are used only for biomass creation).

In the present study, the TDF values of the four Tr-AAs (i.e., alanine, valine, isoleucine, and glutamic acid) were relatively similar (and almost the same for glutamic acid) between the tadpoles feeding on commercial pellets and bloodworms (Table2). These values are also close to the TDF values found in previous studies (Table2). As the commercial pellets and bloodworms are both proteinaceous diets (investigated 11 amino acids can account for 53 and 58 wt.% of the diets, respectively), it could be explained by a scenario in which the following two general processes occur commonly for both feeding experiments: (1) diet-derived amino acids are employed as major resources for the production of energy, and (2) tadpole biomass is composed of the remaining small portion of amino acids with significant enrichment in ^15^N (Fig.6A). A variation in the TDF value between these two feeding experiments is probably caused by different quality of amino acid composition between diets (i.e., homogeneous vs. heterogeneous on the molar ratio of amino acids) and therefore by different deamination flux for each amino acid in tadpoles (Fig.4). In fact, alanine and valine were more abundant in commercial pellets than in bloodworms, assuming that the flux of deamination for these amino acids could be larger for the former diets than the latter diets. This assumption is consistent with the TDF value in these diet experiments (TDF_Commercial pellets >_ TDF_Bloodworms_, Table2). On the other hand, the TDF values of these four amino acids in the tadpoles feeding on boiled rice are relatively small (and significantly small for glutamic acid) compared to those in the tadpoles feeding on commercial pellets and bloodworms (Table2). These results can be explained by a limited metabolic flux into deamination, but a large flux into biomass accrual from the diet-derived amino acids. Here, energy could be supplied satisfactorily from abundant sugar in the boiled rice diet, but limited amounts of amino acids are available from the diets (Fig.6B).

Leucine is more abundant in commercial pellets than bloodworms (Fig.4). However, the TDF value in the tadpoles feeding on commercial pellets (2.0 ± 0.7‰) is smaller than that in the tadpoles feeding on bloodworms (5.6 ± 0.4‰) in the present study as well as than that found in previous studies (Table2). These results may relate to the contribution of catabolic pathway from leucine to isoprenoid lipids such as cholesterol (e.g., Hutson et al. 2005; Mahmud et al. 2005; Kochevenko et al. 2012) when tadpoles feed on commercial pellets. In fact, abundance of cholesterol may be relatively limited in commercial pellets compared to bloodworms because the former are produced from some plant materials (i.e., soybean protein) with no cholesterol. Thus, the small TDF_Leu_ value observed for tadpoles feeding on commercial pellets could be explained when a large portion of leucine was used for this catabolic pathway to cholesterol in tadpole.

## Implications

The TDF values of the Tr-AAs (and glycine in the present study) varied relative to diet type. In other words, diet quality is a potentially important factor influencing the TDF values of the Tr-AAs and glycine in consumer species, especially when diets are lacking in the amount or types of protein needed by a consumer. Our evidence supports this Table2, given that the TDF values of the balanced and high-protein diets were similar to those of previous controlled feeding experiments (particularly similar for glutamic acid and phenylalanine). The predictability of these TDF values appears to be broadly applicable to the trophic ecology of a wide range of organisms, including open-ocean zooplankton (Hannides et al. 2009), deep-sea gorgonian corals (Sherwood et al. 2011), sea slugs that retain photosynthetically active chloroplast (Maeda et al. 2012), Japanese eel larvae (Miller et al. 2012), deep-water ram's horn squid (Ohkouchi et al. 2013), lake gobiid fish (Ogawa et al. 2013), and green turtles (Vander Zanden et al. 2013), as well as the wide variety of fish, arthropods, and gastropods in marine and terrestrial environments (Steffan et al. 2013; Chikaraishi et al. 2014). However, unusually low TDF values have sometimes been reported for certain marine species, such as penguins (Lorrain et al. 2009), large carnivorous fish (Dale et al. 2011; Hoen et al. 2014), sea turtles (Seminoff et al. 2012), jumbo squids (Ruiz-Cooley et al. 2013), and harbor seals (Germain et al. 2013), suggesting that there are unresolved questions relating to diet and consumer physiology, particularly for animals deriving protein from ocean ecosystems. There are also interesting results among holometabolous insects, in which there is no ^15^N-enrichment following metamorphosis (Chikaraishi et al. 2011, 2014), despite the fact that some amount of energy is likely expended during metamorphosis. If the findings in the present study are directly applicable to these wild animals, energy supplied from sugar and/or lipids instead of protein may become a likely explanation for the small TDF values found in wild animals lacking in dietary protein, (as well as the small change in *δ*^15^N among amino acids during insect metamorphosis). Thus, knowledge of the balance among protein, lipid (i.e., fat), and sugar (i.e., carbohydrate) in diets (i.e., PFC balance) will provide a better understanding of the TDF values of consumer species in studied food webs. As protein generally contains much nitrogen relative to sugar and lipids, the C:N ratio of organisms may be useful to know the change in the PFC balance along the food chain or food web. If energy is supplied from sugar or lipids instead of protein (i.e., sugar or lipids are metabolized, but protein remains unmetabolized), the C:N ratio of organisms could be reduced. Moreover, as stable carbon isotopic composition (*δ*^13^C) of sugar and lipids (particularly of a specific carbon atom with a binding site associated with an enzyme during metabolic reactions) may reflect the metabolic flux of the decomposition of sugar and lipids within consumers, we predict that combined analysis of the *δ*^15^N values of amino acids and the *δ*^13^C values of sugar or lipids will be helpful to better evaluate the trophic position of wild animals.

The results of the present study demonstrate that the magnitude of TDF values varies depending on the amount of protein and amino acid composition of the diet. This finding has implications for the interpretation of both wild and cultured animals. In natural systems, wild species may undergo periods in which their trophic position changes due to an exceedingly poor (protein-depleted) diet or simply prolonged starvation. While controlled feeding experiments are fundamental to understanding the dynamics of TDF values in consumer species (e.g., McClelland and Montoya 2002; Chikaraishi et al. 2009; Steffan et al. 2013; Bradley et al. 2014), the TDF values observed may be different from those in wild species if the amino acid composition of diets in feeding experiments is ecologically unrealistic relative to the natural diet. This highlights the need for caution in how controlled feeding studies are designed and conducted. Finally, these results suggest that we can evaluate the appropriate amino acid balance between intake and the minimum required for growth, based on the TDF values of each amino acid. Such information may ultimately be useful to optimize diets for maximal growth and minimal waste within agricultural systems.

## References

[b1] Bender DA (2002). Introduction to nutrition and metabolism.

[b2] Bradley CJ, Madigan DJ, Block BA, Popp BN (2014). Amino acid isotope incorporation and enrichment factors in Pacific bluefin tuna, *Thunnus orientalis*. PLoS One.

[b3] Chikaraishi Y, Kashiyama Y, Ogawa NO, Kitazato H, Ohkouchi N (2007). Biosynthetic and metabolic controls of nitrogen isotopic composition of amino acids in marine macroalgae and gastropods: implications for aquatic food web studies. Mar. Ecol. Prog. Ser.

[b4] Chikaraishi Y, Ogawa NO, Kashiyama Y, Takano Y, Suga H, Tomitani A (2009). Determination of aquatic food-web structure based on compound-specific nitrogen isotopic composition of amino acids. Limnol. Oceanogr. Methods.

[b5] Chikaraishi Y, Ogawa NO, Ohkouchi N, Tayasu I, Koba K, Ohkouchi N (2010a). Further evaluation of the trophic level estimation based on nitrogen isotopic composition of amino acids. Earth, life, and isotopes.

[b6] Chikaraishi Y, Takano Y, Ogawa NO, Ohkouchi N, Tayasu I, Koba K, Ohkouchi N (2010b). Instrumental optimization for compound-specific nitrogen isotope analysis of amino acids by gas chromatography/combustion/isotope ratio mass spectrometry. Earth, life, and isotopes.

[b7] Chikaraishi Y, Ogawa NO, Doi H, Ohkouchi N (2011). ^15^N/^14^N ratios of amino acids as a tool for studying terrestrial food webs: a case study of terrestrial insects (bees, wasps, and hornets). Ecol. Res.

[b8] Chikaraishi Y, Steffan SA, Ogawa NO, Ishikawa FI, Sasaki Y, Tsuchiya M (2014). High-resolution food webs based on nitrogen isotopic composition of amino acids. Ecol. Evol.

[b9] Dale JJ, Wallsgrove NJ, Popp BN, Holland KN (2011). Nursery habitat use and foraging ecology of the brown stingray Dasyatis lata determined from stomach contents, bulk and amino acid stable isotopes. Mar. Ecol. Prog. Ser.

[b10] Décima M, Landry MR, Popp BN (2013). Environmental perturbation effects on baseline δ^15^N values and zooplankton trophic flexibility in the Southern California Current Ecosystem. Limnol. Oceanogr.

[b11] Germain LR, Koch PL, Harvey J, McCarthy MD (2013). Nitrogen isotope fractionation in amino acids from harbor seals: implications for compound-specific trophic position calculations. Mar. Ecol. Prog. Ser.

[b12] Hannides CCS, Popp BN, Landry MR, Graham BS (2009). Quantification of zooplankton trophic position in the North Pacific Subtropical Gyre using stable nitrogen isotopes. Limnol. Oceanogr.

[b13] Hobson KA, Alisauskas RT, Clark RG (1993). Stable-nitrogen isotope enrichment in avian tissues due to fasting and nutritional stress: implications for isotopic analyses of diet. Condor.

[b14] Hoen DK, Kim SL, Hussey NL, Wallsgrove NJ, Drazen JC, Popp BN (2014). Amino acid ^15^N trophic enrichment factors of four large carnivorous fishes. J. Exp. Mar. Biol. Ecol.

[b15] Hutson SM, Sweatt AJ, LaNoue KF (2005). Branched-chain amino acid metabolism: implications for establishing safe intakes. J. Nutr.

[b16] Kochevenko A, Araújo WJ, Maloney GS, Tieman DM, Do PT, Taylor MG (2012). Mol. Plant.

[b17] Lorrain A, Graham B, Ménard F, Popp BN, Bouillon S, van Breugel P (2009). Nitrogen and carbon isotope values of individual amino acids: a tool to study foraging ecology of penguins in the Southern Ocean. Mar. Ecol. Prog. Ser.

[b18] Maeda T, Hirose E, Chikaraishi Y, Kawato M, Takishita K, Yoshida T (2012). Algivore or phototroph? *Plakobranchus ocellatus* (Gastropoda) continuously acquires kleptoplasts and nutrition from multiple algal species in nature. PLoS One.

[b19] Mahmud T, Wenzel SC, Wan E, Wen KW, Bode HB, Gaitatzis N (2005). A biosynthetic pathway to isovaleryl-CoA in myxobacteria: the involvement of the mevalonate pathway. ChemBioChem.

[b20] McCarthy MD, Benner R, Lee C, Fogel ML (2007). Amino acid nitrogen isotopic fractionation patterns as indicators of heterotrophy in plankton, particulate, and dissolved organic matter. Geochim. Cosmochim. Acta.

[b21] McClelland JW, Montoya JP (2002). Trophic relationships and the nitrogen isotopic composition of amino acids in plankton. Ecology.

[b22] Miller MJ, Chikaraishi Y, Ogawa NO, Tamade Y, Tsukamoto K, Ohkouchi N (2012). A low trophic position of Japanese eel larvae indicates feeding on marine snow. Biol. Lett.

[b23] Miura K, Goto AS (2012). Stable nitrogen isotopic fractionation associated with transamination of glutamic acid to aspartic acid: implications for understanding ^15^N trophic enrichment in ecological food webs. Res. Org. Geochem.

[b24] Ogawa NO, Chikaraishi Y, Ohkouchi N (2013). Trophic position estimates of formalin-fixed samples with nitrogen isotopic compositions of amino acids: an application to gobiid fish (Isaza) in Lake Biwa, Japan. Ecol. Res.

[b25] Ohkouchi N, Tuda R, Chikaraishi Y, Tanabe K (2013). Mar. Biol.

[b26] Ohkouchi N, Ogawa NO, Chikaraishi Y, Tanaka H, Wada E (2015). Biochemical and physiological bases for the use of carbon and nitrogen isotopes in environmental and ecological studies. Prog. Earth Planet. Sci.

[b27] Popp BN, Graham BS, Olson RJ, Hannides CCS, Lott M, López-Ibarra G, Dawson TE, Siegwolf RTW (2007). Insight into the trophic ecology of yellowfin tuna, Thunnus albacares, from compound-specific nitrogen isotope analysis of proteinaceous amino acids. Stable isotopes as indicators of ecological change.

[b28] Robbins CT, Felicetti LA, Sponheimer M (2005). The effect of dietary protein quality on nitrogen isotope discrimination in mammals and birds. Oecologia.

[b29] Ruiz-Cooley RI, Balance LT, McCarthy MD (2013). Range expansion of the jumbo squid in the NE Pacific: *δ*^15^N decrypts multiple origins, migration and habitat use. PLoS One.

[b30] Sato R, Kawanishi H, Schimmelmann A, Suzuki Y, Chikaraishi Y (2014). New amino acid reference materials for stable nitrogen isotope analysis. Bunseki Kagaku.

[b31] Seminoff JA, Benson SR, Arthur KE, Eguchi T, Dutton PH, Tapilatu RF (2012). Stable isotope tracking of endangered sea turtles: validation with satellite telemetry and δ^15^N analysis of amino acids. PLoS One.

[b32] Sherwood OA, Iehmann MF, Schuber CJ, Scott DB, McCarthy MD (2011). Nutrient regime shift in the western North Atlantic indicated by compound-specific *δ*^15^N of deep-sea gorgonian corals. Proc. Natl Acad. Sci.

[b33] Steffan SA, Chikaraishi Y, Horton DR, Ohkouchi N, Singleton ME, Miliczky E (2013). Trophic hierarchies illuminated via amino acid isotopic analysis. PLoS One.

[b34] Tsahar E, Wolf N, Izhaki I, Arad Z, Martinez del Rio C (2008). Dietary protein influences the rate of ^15^N incorporation in blood cells and plasma of Yellow-vented Bulbuls (*Pycnonotus xanthopygos*. J. Exp. Biol.

[b35] Vander Zanden HB, Arthur KE, Bolten AB, Popp BN, Lagueux CJ, Harrison E (2013). Trophic ecology of a green turtle breeding population. Mar. Ecol. Prog. Ser.

